# Emphasizing speed or accuracy in an eye-tracking version of the Trail-Making-Test: Towards experimental diagnostics for decomposing executive functions

**DOI:** 10.1371/journal.pone.0274579

**Published:** 2022-09-12

**Authors:** Lukas Recker, Rebecca M. Foerster, Werner X. Schneider, Christian H. Poth

**Affiliations:** 1 Neuro-cognitive Psychology and Center for Cognitive Interaction Technology (CITEC), Bielefeld University, Bielefeld, Germany; 2 Medical School EWL, Bielefeld University, Bielefeld, Germany; University of British Columbia, CANADA

## Abstract

The Trail-Making-Test (TMT) is one of the most widely used neuropsychological tests for assessing executive functions, the brain functions underlying cognitively controlled thought and action. Obtaining a number of test scores at once, the TMT allows to characterize an assortment of executive functions efficiently. Critically, however, as most test scores are derived from test completion times, the scores only provide a summary measure of various cognitive control processes. To address this problem, we extended the TMT in two ways. First, using a computerized eye-tracking version of the TMT, we added specific eye movement measures that deliver a richer set of data with a higher degree of cognitive process specificity. Second, we included an experimental manipulation of a fundamental executive function, namely participants’ ability to emphasize speed or accuracy in task performance. Our study of healthy participants showed that eye movement measures differed between TMT conditions that are usually compared to assess the cognitive control process of alternating between task sets for action control. This demonstrates that eye movement measures are indeed sensitive to executive functions implicated in the TMT. Crucially, comparing performance under cognitive control sets of speed vs. accuracy emphasis revealed which test scores primarily varied due to this manipulation (e.g., trial duration, number of fixations), and which were still more sensitive to other differences between individuals (e.g., fixation duration, saccade amplitude). This provided an experimental construct validation of the test scores by distinguishing scores primarily reflecting the executive function of emphasizing speed vs. accuracy and those independent from it. In sum, both the inclusion of eye movement measures and of the experimental manipulation of executive functions in the TMT enabled a more specific interpretation of the TMT in terms of cognitive functions and mechanisms, which offers more precise diagnoses in clinical applications and basic research.

## Introduction

Diagnostic tests are the cornerstones of psychological assessments for assessing cognitive impairments and guiding clinical treatments. The Trail-Making-Test (TMT, [1]) is among the most widely used diagnostic tests in neuropsychology and related psychological assessments. It consists of two parts in which participants manually connect sequences of targets in a pre-determined order. During part A (TMT-A) participants connect numbers 1 to 25 in ascending order. During part B (TMT-B) participants connect numbers 1 to 13 and letters A to L in ascending order, alternating between the two sets (i.e. 1, A, 2, B, 3 …). In terms of executive functions, correct performance in TMT-B implies a much stronger involvement compared to test version TMT-A [2, 3]. These brain functions enable cognitively controlled behavior [4, 5] and their impairments prevent neurological and psychiatric patients from coping with everyday life [6]. In clinical standard practice, typically a number of different cognitive domains are assessed using different neuropsychological tests. The TMT often constitutes an important part of such an assessment, for instance to diagnose cognitive control impairments of patients suffering from stroke [7], mild cognitive impairment, and dementia [8]. In addition to such clinical applications, the test is widely-used in basic research to study differences in executive functions between healthy individuals [9, 10]. While the TMT offers a practical way to survey individuals’ executive functions, its test scores are based on relatively unspecific compound measures of cognitive performance on a speeded paper-pencil task. Such compound measures can be obtained easily and efficiently and are well suited for cognitive screenings. However, because the measures reflect several different underlying processes collectively, they are hard to interpret in terms of specific cognitive functions and mechanisms. Here, we address this problem by providing a new computer-based version of the TMT that extends the original in two ways: First, it includes eye movement measures that can be interpreted in terms of more specific cognitive functions. Second, it includes an experimental manipulation of participants’ executive functions for action control, namely, their task set as emphasizing speed or accuracy of test performance. This cognitive control function is rarely investigated in neuropsychological tests. We use this manipulation of participants’ task set to evaluate each of the test scores in light of this executive function. As a result, the test scores can now be interpreted either as measures of this executive function or as measures of other cognitive functions (cognitive control and more basic processes). This validates the test scores as measures of the executive function (i.e. providing converging or diverging construct validity), provides a greater conceptual specificity of the interpretation of the scores, and links the scores with mechanistic theories from the literature of experimental and neuro-cognitive psychology. Thus, taken together, by including eye-movement measures and an experimental manipulation of the cognitive control set speed vs. accuracy, the new version of the TMT extends the test’s construct validity and supports a cognitively more specific interpretation for clinical diagnostic decision-making.

Based on accuracy and completion time, the TMT provides a number of different test scores indexing cognitive functions, whereby norms for clinical assessments mostly focus on completion time [3]. Classically, the test is conducted using pencil and paper and participants’ completion times and their errors are manually recorded by the experimenter. If the participant makes an error this is pointed out by the experimenter and the sequence is continued from the last correct stimulus. The completion time for test half A is related to cognitive processing speed [3, 11]. The difference and quotient between the completion times for part A and part B are taken as measures of the ability to shift between task-sets [2, 12]. The completion times and errors in the TMT-B are associated with the individual’s capabilities for visual-spatial processing, working memory, inhibition and behavioral control, and their general cognitive flexibility [2, 12–14]. Therefore, the TMT-B is often used to screen an individual across multiple different executive functions [15]. Critically however, the obtained test scores are relatively unspecific and cannot be interpreted as indices of specific cognitive functions or mechanisms. For instance, interpreting the completion time of the TMT-A as a measure of “cognitive processing speed”, is rather descriptive and does not provide insight into possibly affected cognitive functions (e.g. localization of action targets, movement planning, etc.) or cognitive mechanisms (e.g., attention mechanisms performing the localization of action targets, etc.). One way to address this problem is to include eye-tracking measures into the test. Most of these measures are based on saccadic eye movements and intervening fixations. There is a wealth of neurophysiological [16–18] and cognitive research [19–21] on the cognitive functions implicated in saccadic eye movements and on the neuro-cognitive mechanisms fulfilling these functions [22–24]. As a result, currently there exist a number of theories and models enabling a specific interpretation of saccade-related performance measures.

To increase the conceptual specificity and better depict the determinants of performance in the test, we extend the TMT by including a number of different eye movement measures. First, fixation durations enable the closer examination of the processing of visual stimuli. The time participants spend fixating on objects in large parts parallels the time needed to extract the necessary information for subsequent actions and planning [25]. Thus, fixation durations describe in greater detail the ongoing cognitive mechanisms during task performance. Second, the size of executed eye movements represented by saccade amplitudes can provide insights into strategic cognitive processes employed by participants [26]. For instance, participants could either scan their environment using large- or small-scale eye movements. Furthermore, as indicated by previous studies both fixation durations and saccade amplitudes may be determined by stable endogenous factors of individuals and thus could present a useful tool to differentiate between persons [27–29]. Third, the overall number of fixations can indicate applied scanning strategies [30] and can for instance imply the number of times participants shift their attention during the task. Previous studies of the TMT which included eye-tracking indicated differences in the number of fixations across test halves [31–33] and stability of saccade amplitude and fixation duration [33].

The available measures provided by eye tracking further extend to the connection of eye and hand movements in the task. Since the task requires the participants to consecutively connect targets via lines, measures that examine and describe more closely the role and relationship of eye movements during sequences of manual actions can be determined. These measures include characteristics of fixation types [20, 34] or the interplay of the eyes and the hands [35, 36]. In particular, based on the current position in the sequence fixations can be categorized as either falling on the current target, i.e. guiding fixations, or past and future targets, i.e. searching fixations. Guiding fixations are assumed to play a central role in coordinating eye and hand movements. Usually, prior to a hand movement to a target object (e.g., reaching for a glass of water), the eyes land on the target, allowing more efficient arm movements to the foveated location [20, 37]. In our eye tracking based TMT version, a fixation on the current clicking target object (e.g., on number 5) should contribute to more precise clicking on this object. Moreover, searching fixations are required when the target object for hand movements has not been found and foveated yet [37]. For the TMT, prior to clicking on the current target (e.g. after a preceding click on number 4, the current search target should be number 5), searching fixations at potential target locations are made and the search process stops once the current target is found (e.g., number 5). Furthermore, the temporal coupling of eyes and hands is illustrated in the eye-hand span, the time between the fixation on an action target and the subsequent action. This measure is often applied to study differences between novices and experts, e.g. in reading music or other sensory-motor tasks [36, 38, 39], and thus describes expertise in and difficulty of a task more closely. Lastly, the length of the scanpath can describe how efficient participants are in covering the search area and provide an additional indicator of the effectiveness in gathering information. Taken together, these measures grant additional insights into basic neuro-cognitive functions [20, 37, 40, 41] especially into visual selection processes and thus present a valuable source of complementary information when understanding performance in the TMT.

So, including eye-tracking in the TMT should improve the validity and conceptual specificity of the test by providing additional measures interpretable in terms of their respective cognitive functions. This has proven helpful not only for the TMT but a wide range of neuropsychological tests before (e.g. Corsi blocks, [42]; spatial working memory, [43]). Still, relating test scores to differences between the test halves leaves a lot of room for interpretation since both differ greatly in their cognitive demands and in turn their underlying cognitive functions. However, this problem can be addressed by combining the TMT with an experimental manipulation of participants’ task set whose influence on executive and other cognitive functions more generally has been thoroughly established in the neuro-cognitive literature. One type of fundamental task set that regulates performance on any given cognitive task is the one trading-off the speed against the accuracy of task-related actions [44, 45]. When participants employ a task set emphasizing speed, actions become faster at the expense of a lower accuracy and more errors, whereas when they employ a task set emphasizing accuracy, actions becomes slower at the benefit of a higher accuracy and fewer errors (for a review, see [45]). This speed-accuracy trade-off seems to reflect one of the few general laws in psychology, as it is evident in a wide variety of cognitive tasks (e.g., decision-making, [46], visual search [26, 47], memory retrieval [44]), and even extends to other species [48]. Including this manipulation to the TMT targets a set of executive functions that are fundamental to human intelligent behavior, as they are implicated in the dynamic adaptation of behavior to current task demands [4, 49]. Specifically, these functions consist in dynamically adopting specific cognitive task sets, representations of task demands which govern the cognitive processes underlying task performance [50]. Comparing TMT performance under these experimentally manipulated conditions allows to assess how each test score is affected by this manipulation, and the variation of test scores due to this effect can then be compared to the interindividual variation within the test scores. Test scores that primarily reflect the speed-accuracy task set vary more strongly due to the manipulation, whereas test scores that do not primarily reflect this construct vary more strongly due to interindividual differences. Therefore, the focus of including the speed-accuracy manipulation in the test lies not in specifying the exact effects of focusing on either one task set but in enabling connections of test scores to the underlying cognitive control function. In other words, using this approach, one can use a theory-based manipulation of a cognitive construct to test whether it is reflected or not reflected in a given test score of the TMT. In this way, the approach extends the test scores’ validity by offering an experimentally-based construct validity, which allows a more specific interpretation of the test score in terms of the cognitive functions and mechanisms given by the neuro-cognitive theories the manipulation is based on. To the best of our knowledge, this study is the first that aims to use speed-accuracy instructions in this way.

In the present study, we aimed to increase conceptual specificity with which the test scores of the TMT can be interpreted in terms of cognitive functions and mechanisms. To this end, we provide a computer-based version of the TMT that extends the original with eye movement measures allowing a more specific examination of reflected cognitive processes and with an experimental manipulation of participants’ executive functions for action control, namely, their task set as emphasizing speed or accuracy of test performance. We test the new version of the TMT in an experiment comprising the TMT-A and the TMT-B.

First, the additionally included eye tracking measures were investigated with regard to differences between TMT-A and TMT-B. If a test score differed between test half A and B, then the underlying cognitive function could provide additional information about the performance related to known differences in demands of the test halves. However, since differences between TMT-A and TMT-B could still reflect a number of different cognitive functions in either test half we aimed to furthermore link test scores to processes involved in action control. If test scores were affected by the speed and accuracy instructions, then these scores should reflect the state of the current task set. This effect could then provide the link to active processes in action control when emphasizing either speed or accuracy.

In addition, for each test score we could compare the variance stemming from interindividual differences with the variance induced by the experimental manipulation of participants’ speed vs. accuracy task sets. If a test score primarily reflected the speed vs. accuracy task set, then its variance should be dominated by the task set manipulation and not by interindividual differences. In contrast, if a test score primarily reflected a cognitive construct different from the speed vs. accuracy task set, then the variance of this score should be dominated by interindividual differences and not by the task manipulation.

## Methods

### Participants

Sixty-two healthy participants (43 female, 19 male) from Bielefeld University were paid to take part in the experiment, two of which had to be excluded due to missing data. They were between 18 and 28 years old (*M* = 21.71, *SD* = 2.24). All of them were right-handed and reported normal or corrected-to-normal vision. Before the experimental procedure, participants gave written informed consent. The experiment was conducted in accordance with the ethical guidelines of the German Psychological Association (Deutsche Gesellschaft für Psychologie) and was approved by the ethics committee at Bielefeld University.

### Apparatus and stimuli

The experiment was conducted in a dimly lit room, where participants sat down in front of a 19 inch monitor (ViewSonic G90fb) with a resolution of 1024 x 768 pixels (corresponding to physical dimensions of 36 cm x 27 cm (width x height) and a refresh rate of 100 Hz (pre-heated for at least 5 min, [51]). Their head was stabilized on a chin rest 71 cm from the screen. Movements of their right eyes were recorded at 1000 Hz, using the desktop-mounted eye-tracker EyeLink 1000 (SR Research, Ontario, Canada). During the experimental trials, they navigated the cursor with a computer mouse (Logitech RX250), whose positions were sampled at 100 Hz (time-locked to screen refresh). Stimulus presentation was controlled by the Experiment Builder software (SR Research, Ontario, Canada).

Stimuli consisted of black letters and/or numbers surrounded by a black, unfilled circle. In version A of the stimulus set, numbers 1 to 25 constituted the target stimuli. In version B of the stimulus set, numbers 1 to 13 and letters A to L constituted the target stimuli. In both versions, they were presented in Arial with the font size set to 16 pt (approximate height 0.59 ° va). The surrounding circle measured 1.35 ° va diameter, its line width was set to 5. The mouse cursor was represented by a small black, filled circle measuring 0.42 ° va diameter. Stimuli were presented on a uniform grey background (30 cd/m^2^, measured with a MAVOLUX-digital luminance meter). Stimulus positions were the same for all participants within TMT-A and -B respectively. That is, each participant completed the same spatial arrangement in TMT-A and TMT-B trials, while the order of the speed or accuracy instructions was counterbalanced across arrangements within the blocks. The positions consisted in points scattered around the center points of a randomly chosen field from a 5-by-5 grid spanning the entire screen (i.e. with a field size of 5.72 x 4.32 (width x height) ° va). However, they were not specifically matched across test halves. Sounds were provided as feedback for the participants’ answers. A high-pitched sound (53 dB(A), measured with a Mengshen MS-M80A) played for 70 ms indicated a hit, a low-pitched sound (46 dB(A)) played for 100 ms indicated a miss.

### Procedure

**Fig 1:**
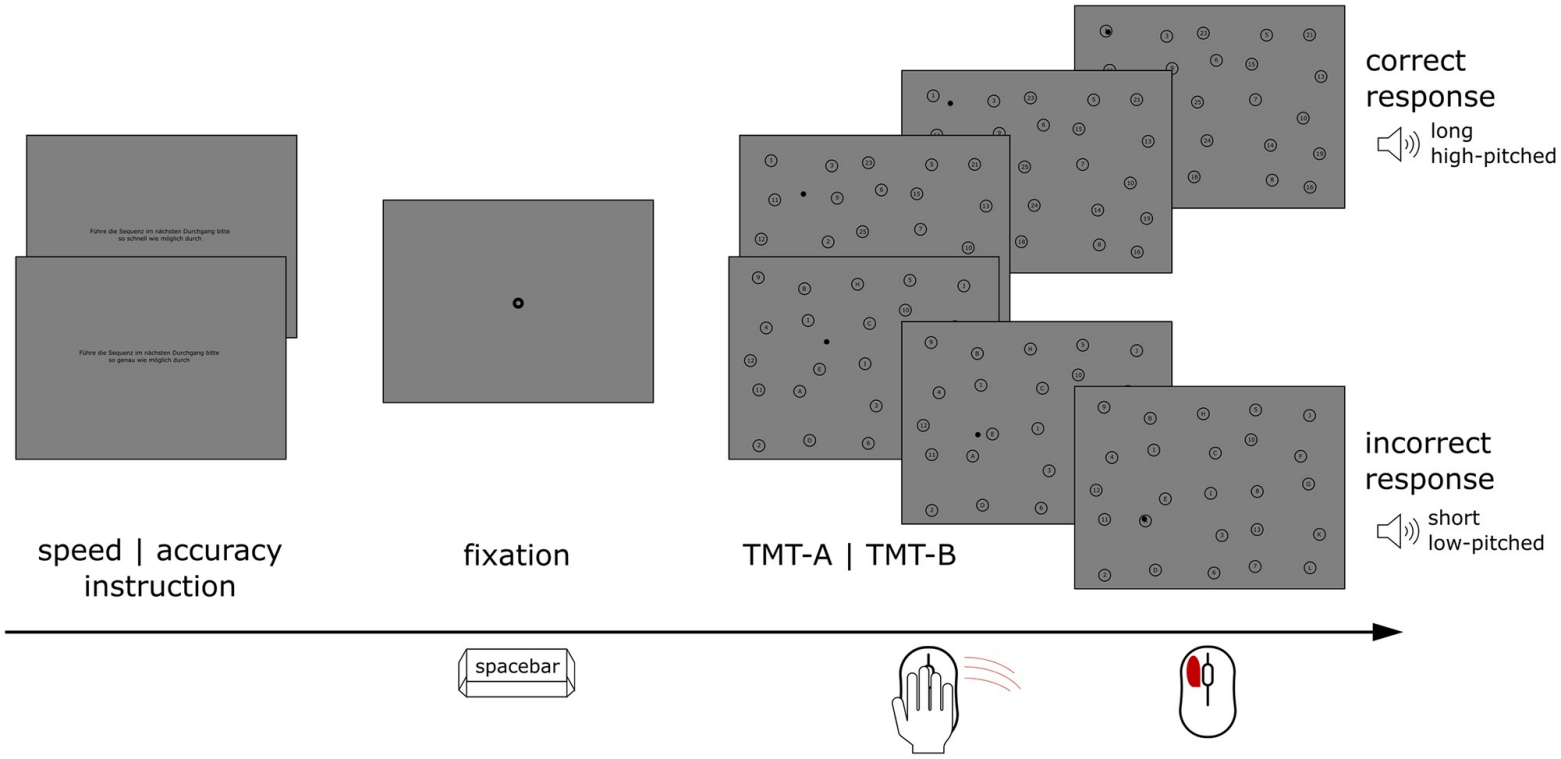
Trial procedure. Time course of a trial in this version of the TMT. Participants were first instructed to hit targets in the following trial either as fast (speed) or as central (accuracy) as possible. After a fixation display, they started the TMT sequence by pressing the space bar. Participants clicked the sequence of numbers (TMT-A) or numbers and letters (TMT-B) in ascending order while their eye movements were recorded. Correct answers were indicated by a high-pitched sound, wrong answers were indicated by a low-pitched sound. The black dot indicates the current position of the mouse.

The experiment was divided into two blocks (A and B), each consisting of one training and two experimental trials. Each block started with a written instruction on the screen after which the eye tracker was calibrated using a 9-point calibration and validation procedure. In block A (i.e. TMT-A) participants’ task was to click the target stimuli, numbers 1 to 25, in ascending order (cf. Fig 1 for a depiction of the trial procedure). In block B (i.e. TMT-B) participants’ task was to click the target stimuli, numbers 1 to 13 and letters A to L, in ascending order, alternating between the two sequences (e.g. 1, A, 2, B, 3, …, 13). Different from the paper-pencil version of the test, targets were not connected via lines. This also meant that targets next to each other in the sequence did not need to be in adjacent circles in the stimulus display and intermediate targets could be moved across by the participant. Participants first completed the training trial with a reduced number of stimuli (8 targets). The experimental trials then were each preceded by a short, written instruction, introducing the speed or accuracy condition of the following run. In the speed condition, participants were asked to complete the sequence as fast as possible. In the accuracy condition, participants were asked to hit the targets as central as possible. Either way, responses were counted as correct if they fell within the circle surrounding the number/ letter. A correct response was followed by a high-pitched sound, an incorrect response was followed by a low-pitched sound. Participants could only move on to the next target in sequence if they successfully hit the previous one. A centrally presented black ring, serving as a fixation stimulus preceded each trial (0.45 ° va outer circle, 0.11 ° va inner circle). Participants could start each trial by pressing the space bar. Altogether, the experiment comprising the two blocks and three trials each was completed within 10 minutes. The order of the blocks and the trial instructions within the blocks was counterbalanced across participants.

### Dependent variables

To detect fixations, saccades and blinks, the algorithm of the EyeLink 1000 was used. Saccades and fixations were detected online using a velocity threshold of 30 ° va × s^-1^ and an acceleration threshold of 8000 ° va × s^-2^. Fixations were merged if they were less than 1 ° va apart. The analysis includes the following basic dependent variables: the trial duration provides the measure for the completion time in each trial, it was computed as the time between participants signal to start (hitting the space bar) and the successful completion of the last target (mouse click on target); the fixation durations, saccade amplitudes, and number of fixations as provided by the online detection algorithm of the EyeLink 1000. Furthermore, the following dependent variables were added: the number of searching fixations, i.e. fixations visiting future or past targets., guiding fixations, i.e. fixations visiting the present target [20, 40] scanpath length, and eye-hand span, i.e. the time between a fixation on the target and a consequent action. To define a fixation as either searching or guiding, the position of the fixation must fall within a circle with 3.25 ° va diameter around the target center. To calculate the eye-hand span the timestamp of the first guiding fixation is taken and compared to the timestamp of the matching click. Resulting spans indicate the time by which the eyes lead the hands [36].

## Results

### Statistical analyses

The data and fully documented analysis code have been made publicly available at the Open Science Framework and can be accessed at: https://osf.io/E4UR5/. Data analysis was done in R (Version 4.1.3 [52]). We divided the data analysis into two steps: First, we examined the effects of the speed-accuracy instructions on the outcome measures provided in this TMT. We therefore used linear mixed-effect models with subjects as random factors employing the lme4 package (Version 1.1–28 [53]). Fixed effects could comprise the within subject factors test type (TMT-A vs. TMT-B) and instruction (accuracy vs. speed), as well as their interaction term. Factors are dummy-coded for the analyses. Effects of test type are always in reference to factor level TMT-A. Effects of instruction are always in reference to factor level accuracy. Estimates of p-values were provided by the lmerTest-package (Version 3.1–3 [54]) using a significance-criterion of p < .05, however because estimation of p-values in mixed models is not without issues, we focus more on the confidence intervals of the estimates. The data of two participants were excluded from the analysis due to missing values. Additionally, we report the best fitting models which means for some dependent variables up to two additional data sets were excluded. However, this did not alter results.

Second, we developed a procedure to determine the degree to which dependent variables are dominated by the effects of the task set manipulation emphasizing speed or accuracy in TMT-A and TMT-B or interindividual variability respectively. Therefore, we compared the influence of the speed-accuracy manipulation with the interindividual variability, i.e. the fixed and the random effects, in the data. The comparison of fixed and random effects was achieved by calculating the quotient of the Bayes Factor (BF_10_, BayesFactor-package; [55]) of a model comprising only the fixed effects structure divided by the Bayes Factor of a model comprising only the random effects. The result indicated whether the fixed effects-only or the random effects-only model was more likely to find the given data compared to an intercept-only model. Additionally, we calculated marginal R-squared and conditional R-squared values for the model [56]. These values represent estimates describing the variance that is explained by the model’s fixed effects (marginal) and the combination of fixed and random effects (conditional).

**Fig 2:**
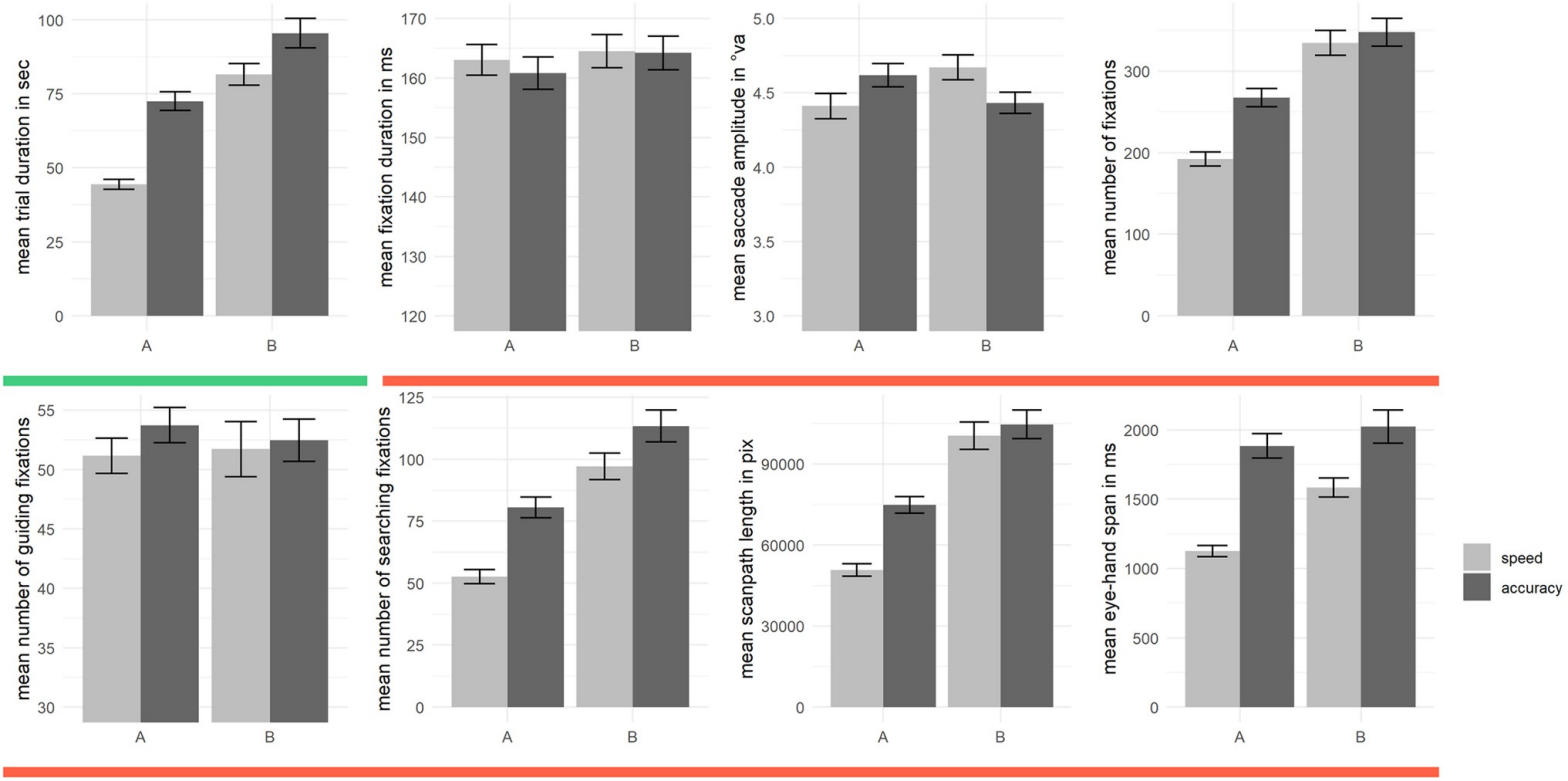
Test scores in TMT-A/-B under speed/accuracy instructions. Bar plots for the dependent variables across all conditions (test halves A and B; speed and accuracy instruction). Error bars represent the standard error of the mean. The red line underneath the plots indicates variables representing eye movement control measures. The green underneath the plots indicates variables representing classic TMT measures, i.e. trial duration.

**Fig 3:**
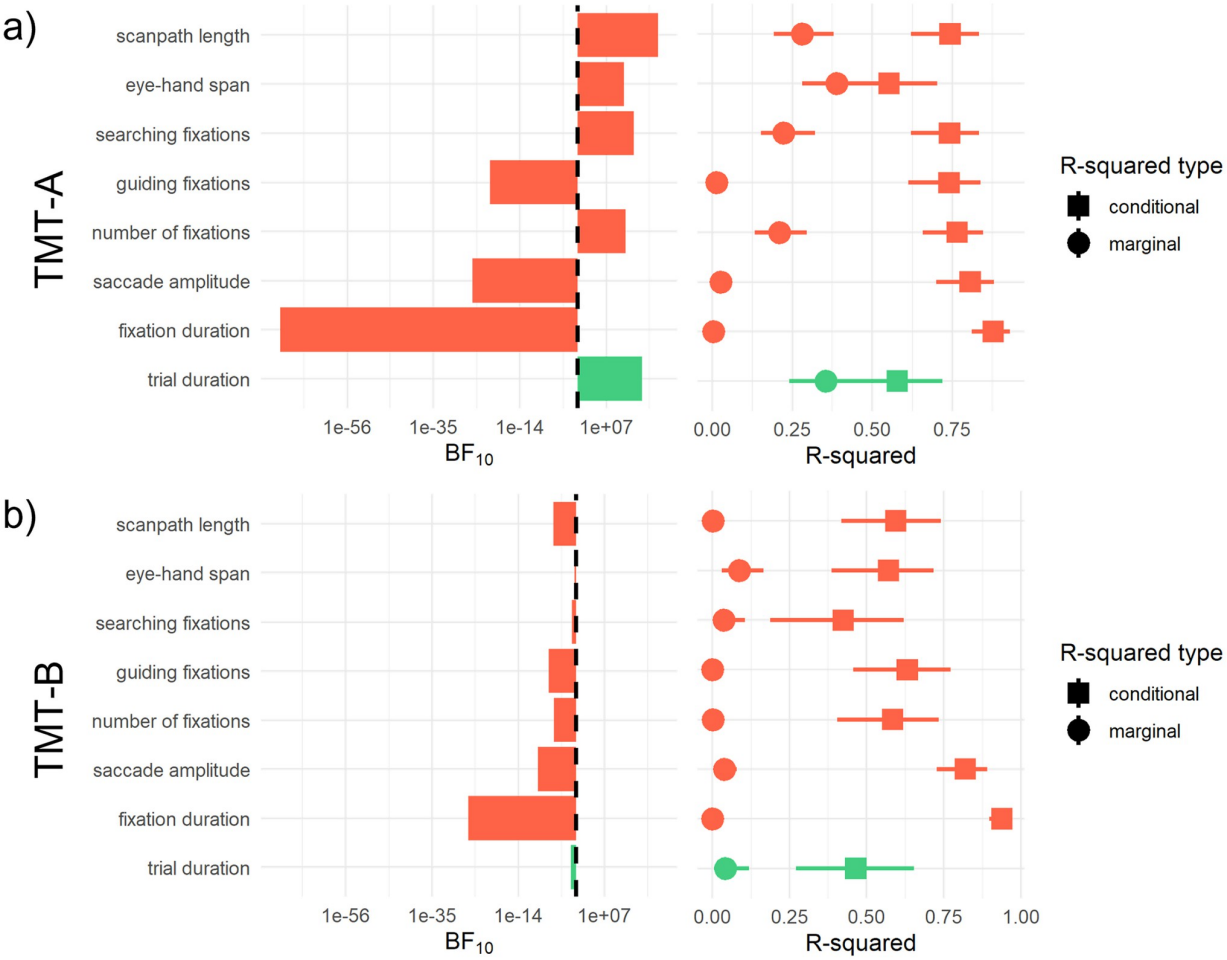
Variables dominated by the individual variability vs. the speed-accuracy manipulation. Results of the analyses for R-squared values and the Bayes Factor comparison of fixed versus random effects in the model for each examined dependent variable in TMT-A (Fig 3a) and TMT-B (Fig 3b). Error bars in the R-squared plot represent bootstrapped 95% C.I. All variables related to eye movement control are plotted in red. All variables related to classic TMT measures, i.e. trial duration, are plotted in green.

We repeated the described procedure for each dependent variable. The results of the analyses are described in the following. The first analysis step is depicted in Table 1 and Fig 2. Additionally, we provide a table including the descriptive statistics illustrated in Fig 2 in the supplementary material (see S1 Table). The second analysis step is depicted in Fig 3. A commented version of the analysis code further describing the newly applied method is included in the OSF repository.

**Table 1:** Results of the linear mixed-effect models.

DV	*Fixed Parts*				*Random Parts*		
	*Betas*						
	(Intercept)	test type	instruction	test type × instruction	N_group_	Observations	SD_Intercept_
trial duration	72.55 [65.58–79.52]	22.97 *** [15.37–30.58]	-28.18 *** [-35.58 –-20.57]	14.20 * [3.42–24.98]	58	231	17.22
fixation duration	160.80 [155.45–166.15]	3.40 ** [1.14–5.65]	2.22 [-0.03–4.48]	-1.90 [-5.08–1.29]	58	232	19.85
saccade amplitude	4.62 [4.46–4.77]	-0.18 ** [-0.31 –-0.06]	-0.21 ** [-0.33 –-0.08]	0.45 *** [0.27–0.63]	59	236	0.50
n fixations^a^	5.54 [5.45–5.62]	0.25 *** [0.17–0.33]	-0.33 *** [-0.41 –-0.26]	0.30 *** [0.19–0.40]	59	236	0.26
n guiding fixations	53.73 [50.20–57.26]	-1.27 [-4.42–1.88]	-2.58 [-5.73–0.57]	1.83 [-2.62–6.28]	59	236	10.74
n searching fixations^a^	4.31 [4.20–4.42]	0.34 *** [0.23–0.45]	-0.43 *** [-0.54–-0.32]	0.26 ** [0.11–0.41]	59	236	0.30
eye-hand span^a^	7.48 [7.39–7.57]	0.05 [-0.04–0.14]	-0.49 *** [-0.58–-0.40]	0.27 *** [0.15–0.40]	57	228	0.23
scanpath length^a^	11.17 [11.09–11.27]	0.31 *** [0.23–0.39]	-0.39 *** [-0.49 –-0.31]	0.35 *** [0.24–0.47]	59	236	0.27

DV = dependent variable;

^a^ = marked variables were log-transformed;

n = number of …; SD = standard deviation; Numbers in squared brackets represent 95% confidence intervals; p-value estimates are indicated by

* < .05,

** < .01,

*** < .001 (Intercepts all had p < .001)

### Dependent variables

#### Trial durations

Like in all versions of the TMT the trial duration was overall longer in TMT-B (test type: *b* = 22.974, *SE* = 3.879, *t* = 5.921, *p* < .001). Participants were faster under speed-instructions (*b* = -28.177, *SE* = 3.879, *t* = -7.263, *p* < .001). This effect was less pronounced in TMT-B (*M*_diff_ = 14.09 *s*, *SD* = 35.26 *s*) than it was in TMT-A (*M*_diff_ = 28.18 *s*, *SD* = 21.82 *s*; *b* = 14.200, *SE* = 5.501, *t* = 2.581, *p* < .05).

The additional analyses indicated a prevailing effect of the experimental speed-accuracy manipulation over interindividual variability in the TMT-A but not the TMT-B. The Bayes Factor comparison strongly favored the fixed effects-only model (BF_10_ = 4.07 × 10^11^) in the TMT-A. In the TMT-B the random effects-only model was favored (BF_10_ = 0.07). The R-squared values supported these results (TMT-A: *R*_*m*_^*2*^ = .355 [.240, .463]; *R*_*c*_^*2*^ = .578 [.408, .720]; TMT-B: *R*_*m*_^*2*^ = .043 [.005, .120]; *R*_*c*_^*2*^ = .465 [.270, .652]) indicating predominant influences of the speed-accuracy manipulation in the TMT-A but not the TMT-B.

#### Fixation duration

Fixation durations were slightly longer in TMT-B (*M* = 164.36 ms, *SD* = 21.31 ms) than in TMT-A (*M* = 161.91 ms, *SD* = 20.11 ms), although the effect was small (*b* = 2.448, *SE* = 0.817, *t* = 2.996, *p* <. 01). Also, this was not influenced by speed or accuracy instructions. Generally, the instructions had no effects on fixation durations (all *b*s < 2.224, *t*s < 1.935, *p*s > .05).

The second part of our analysis for the fixation durations of participants revealed a strong influence of interindividual variability in either half of the test. The comparison of the Bayes Factors underlined the presenting picture, suggesting strong evidence for the random effects-only model in the TMT-A, BF_10_ = 1.75 × 10^−18^, and the TMT-B, BF_10_ = 6.99 × 10^−27^. Accordingly, the marginal R-squared of the final model was close to 0 in both test halves (TMT-A: *R*_*m*_^*2*^ = .003 [.000, .015]; TMT-B: *R*_*m*_^*2*^ = .000 [.000, .003]), while the conditional R-squared was much greater (TMT-A: *R*_*c*_^*2*^ = .878 [.810, .929]; TMT-B: *R*_*c*_^*2*^ = .937 [.896, .962]).

#### Saccade amplitude

Results for participants’ saccade amplitude suggested influences from all experimental factors. However, all effects were of small magnitude as evident by the confidence intervals close to 0 (see Table 1).

This pattern further established in the second part of the analysis. Bayes Factor comparisons, too, favored the random effects-only model in the TMT-A, BF_10_ = 9.27 × 10^−11^ and the TMT-B, BF_10_ = 6.00 × 10^−10^. Taken together with the calculations for the R-squared estimates, this suggested a predominant influence of interindividual variability (TMT-A: *R*_*m*_^*2*^ = .027 [.008, .061]; *R*_*c*_^*2*^ = .806 [.699, .881]; TMT-B: *R*_*m*_^*2*^ = .040 [.014, .079]; *R*_*c*_^*2*^ = .819 [.726, .890]).

#### Number of fixations

The model for participants’ number of fixation was fitted to log-transformed values, since they indicated a better fit than the original values. Both the test type (*b* = 0.250, *SE* = 0.039, *t* = 6.460, *p* < .001), the instruction (*b* = -0.331, *SE* = 0.039, *t* = -8.557, *p* < .001) and their interaction (*b* = 0.297, *SE* = 0.055, *t* = 5.430, *p* < .001) influenced the number of fixations. Since participants also spent more time on the respective task, these results were in line with the results of the trial duration, i.e. they performed more fixations in TMT-B, less during speed instructions, while the latter effect was less pronounced in TMT-B than in TMT-A.

Analyzing the number of fixations applying the second analysis method comparing the Bayes Factors for fixed effects-only and random effects-only models, the results indicated a better fit of the fixed effects-only model in the TMT-A, BF_10_ = 7190. Similar to the trial durations, this was not the case in the TMT-B, BF_10_ = 4.92 × 10^−6^. R-squared values supported these results, revealing that the fixed effects accounted for .211 [.133, .296] proportion of the variance, fixed and random effects for .765 [.658, .846] in the TMT-A. In the TMT-B fixed effects accounted for .002 [.000, .027] proportion of the variance, fixed and random effect for .584 [.404, .733]. Thus, the number of fixations was predominantly determined by the speed-accuracy instructions in the TMT-A but not the TMT-B.

#### Number of guiding fixations

Results for the number of guiding fixations mirrored the results for the saccade amplitudes. Here, all confidence intervals for the estimates of the factors included 0. Therefore, we found no differences in participants’ number of guiding fixations depending on test type or instruction.

The second part of our analysis revealed that the comparison of the Bayes Factors supported the evidence for the importance of interindividual variability compared to the task influences in both TMT-A, BF_10_ = 1.24 × 10^−9^, and TMT-B, BF_10_ = 2.69 × 10^−7^. Estimates of the R-squared values were .013 [.001, .043] for the marginal and .740 [.613, .838] for the conditional R-squared in the TMT-A and .001 [.000, .019] for the marginal and .631 [.456, .770] for the conditional R-squared in the TMT-B respectively. Similarly to fixation durations and saccade amplitudes, the number of guiding fixations were predominantly determined by interindividual variability in both test halves.

#### Number of searching fixations

The model for the number of searching fixations indicated influences for both factors (test type: *b* = 0.340, *SE* = 0.055, *t* = 6.152, *p* < .001; instruction: *b* = -0.432, *SE* = 0.055, *t* = -7.813, *p* < .001) and their interaction (*b* = 0.258, *SE* = 0.078, *t* = 3.305, *p* < .01), similar to the general number of fixations. Here too, we log-transformed the dependent measure to assure the better model fit. Taking together the results of the overall fixations and the separate fixation types, the pattern indicates that the change in overall number of fixations stems from influences on the number of searching fixations.

The Bayes Factors calculated for the comparison of fixed and random effects influence in the second part of our analysis, came out in favor of fixed effects impact, BF_10_ = 4.73 × 10^4^ in the TMT-A and in favor of the random effects impact, BF_10_ = 0.12 in the TMT-B. We obtained R-squared values of *R*_*m*_^*2*^ = .222 [.151, .322] and *R*_*c*_^*2*^ = .742 [.622, .834] in the TMT-A and *R*_*m*_^*2*^ = .038 [.004, .105] and *R*_*c*_^*2*^ = .424 [.188, .619] in the TMT-B. Akin to the general number of fixations, the results present a more divided picture, but stress the effects of the experimental task manipulation on the number of searching fixations in the TMT-A.

#### Eye-hand span

Results of the model for the eye-hand span indicated that the instruction (instruction: *b* = -0.493, *SE* = 0.049, *t* = -10.088, *p* < .001) and the interaction between test type and instruction (*b* = 0.273, *SE* = 0.057, *t* = 4.802, p < .001) had an impact on the temporal distance between participants fixation on a target and the following click. For the instruction the eye-hand span was shorter under speed- than under accuracy instructions (M_speed_ = 1.35 s, SD_speed_ = 0.48 s; M_accuracy_ = 1.95 s, SD_accuracy_ = 0.79 s). Furthermore, this effect was smaller for eye-hand spans in TMT-B. Descriptively, the eye-hand span was shorter in TMT-A (M = 1.50 s, SD = 0.64 s) than in TMT-B (M = 1.80 s, SD = 0.76 s). However, the confidence interval of the estimate included 0.

The Bayes Factor comparisons for the eye-hand span suggested an advantage of the fixed effects-only model over the random effects-only model in the TMT-A, BF_10_ = 1.13 × 10^13^. But again, turned around in the TMT-B, BF_10_ = 0.55. The resulting R-squared values for the model were *R*_*m*_^*2*^ = .389 [.282, .511] and *R*_*c*_^*2*^ = .552 [.401, .703] in the TMT-A, and *R*_*m*_^*2*^ = .086 [.030, .165] and *R*_*c*_^*2*^ = .570 [.386, .716] in the TMT-B. These results suggest a predominant influence of the experimental task manipulation on the eye-hand span in the TMT-A only.

#### Scanpath length

Finally, for the scanpath length we found comparable results to the trial duration and number of fixations. The model fitted to the log-transformed values included both factors test type, *b* = 0.310, *SE* = 0.043, *t* = 7.156, *p* < .001 and instruction, *b* = -0.395, *SE* = 0.040, *t* = -9.750, *p* < .001, as well as their interaction, *b* = 0.354, *SE* = 0.057, *t* = 6.816, *p* < .001. Participants scanpath length fitted the pattern that more time and more fixations were spent in TMT-B, while speed-instructions shortened the scanpath length, especially in TMT-A.

The second part of our analysis further established the reoccurring picture. Again, the comparison of the Bayes Factors suggested a greater influence of experimental task manipulation on the length of the scanpath than interindividual variability, BF_10_ = 2.95 × 10^7^ but only in TMT-A. The Bayes Factor in the TMT-B suggested a greater influence of individual variability on the length of the scanpath than of the experimental task manipulation, BF_10_ = 3.24 × 10^−6^. The resulting R-squared values were *R*_*m*_^*2*^ = .280 [.192, .379] and *R*_*c*_^*2*^ = .743 [.621, .833] in the TMT-A, and *R*_*m*_^*2*^ = .003 [.000, .030] and *R*_*c*_^*2*^ = .593 [.418, .739] in the TMT-B.

## Discussion

Diagnostics tests provide the tool for neuropsychological assessment to identify cognitive impairments and profile the strengths and weaknesses of individuals. As one of the most popular tests in neuropsychological diagnostics, the TMT is frequently applied to survey participants’ processing speed and executive functions. Despite the test’s widespread use, the provided test scores portray rather unspecific compound measures of cognitive functions since they are based on global test completion times. Here, we provided a new version of the TMT aiming to increase the test scores’ conceptual specificity and their interpretation in terms of cognitive processes and mechanisms. To this end, we included additional eye tracking measures to the test and added an experimental manipulation of participants’ task set. Contrasting the eye-tracking-based test scores from the different test halves revealed which of these measures were sensitive to the compound measure of task set switching of the classic TMT. As the cognitive processes captured by these measures are better understood in terms of neurophysiology and cognition [16, 19, 57], they can aid interpretation by revealing specific component processes of the broader compound measure of the classic TMT. Furthermore, for each test score we compared the interindividual variance with the variance introduced by the experimental manipulation of emphasizing speed or accuracy. This comparison allowed us to dissociate test scores from or associate test scores with the experimental manipulation and evaluate them in relation to the cognitive constructs linked with the speed vs. accuracy task set. Taken together, this demonstrated how expanding the TMT with experimentally-based resources can improve the understanding of examined test scores and validate them by providing links to cognitive processes and mechanisms grounded in cognitive theory and experimental basic research.

### Eye-tracking measures in the TMT

The new version of the TMT included two distinct additions: added test scores provided by eye-tracking and a manipulation of participants’ task set, emphasizing speed or accuracy. The aim of the inclusion of eye-tracking measures in the TMT was to increase the specificity of provided test scores in terms of enabled interpretations considering cognitive functions associated with the execution of eye movements. *Fixation durations* and *saccade amplitudes* were relatively stable across test halves and speed-accuracy instructions. Neither the numerous differences between TMT-A and -B in affected cognitive functions [2], nor the additional manipulation of participants’ task set had substantial impact on the duration of their fixations or the magnitude of their eye movements. Due to the different spatial arrangement of targets in the test halves, we have to be cautious when interpreting measures that are closely related to spatial components of the task like the saccadic amplitude. However, since the stability of fixation durations and saccade amplitudes parallels previous work examining these measures across different tasks [27, 28, 58] and the TMT [33] we are confident that these measures might reflect stable interindividual differences also in the context of this TMT. The *number of fixations*, increased from TMT-A to TMT-B and interacted with the speed-accuracy instructions. Previous investigations of eye movements in the TMT which found differences in the number of fixations across test halves [31–33] provided consistent results. Performance in the TMT-B is thought to reflect many underlying cognitive processes including behavioral control and inhibition [12, 13]. An increased number of fixations aids interpreting differences between test halves by demonstrating concrete effects of the additional demands on participants behavior in terms of their eye movement control. The *number of searching fixations* and the *scanpath length* both increased from TMT-A to TMT-B and also interacted with the speed-accuracy instructions. Especially the scanpath length is heavily influenced by characteristics of the search display like the location of stimuli. However, the magnitude of differences in the scanpath lengths between TMT-A and TMT-B together with the paralleling results from trial durations and the general number of fixations increasing from part A to B suggest effects beyond influences of spatially different arranged stimuli. Different cognitive demands in the test halves are therefore also reflected in characteristics of the scanpath and the number of searching fixations The *eye-hand span*, i.e. the temporal distance between participants’ fixation on a target and the following click, did not differ between TMT-A and TMT-B but was affected by the speed-accuracy instructions. Up until now, no measures specific to action sequence tasks, like the number of different fixation types or the temporal relation between eye and hand movements, were included in a study investigating the TMT. Studies examining the effects of affordances [41] and learning of action sequences [40], suggest that these kinds of measures might be sensitive to task-related differences. Our results for these eye movement measures in part support this assumption. We found that the number of searching fixations, the eye-hand span, and the scanpath length were affected by the current task set. The *number of guiding fixations* in contrast was relatively stable across both test halves and the speed-accuracy instructions. Also considering the results on the overall number of fixations which can be attributed to differences between searching but not guiding fixations suggests an important role of search related processes in the TMT and enhances the understanding of underlying cognitive processes in that way. Taken together, the included eye-tracking measures can increase the specificity of interpretations of test scores in the TMT in two ways. Test scores varying with the specific demands of the task contain information about the established executive functions underlying the TMT. Furthermore, test scores that are stable across the task can help narrow down which aspects of the executive functions are influenced and which are not. It should be noted that the spatial arrangement and thus the length of the overall trail participants had to complete was randomized and thus not separately controlled for between TMT-A and TMT-B. Therefore, test scores particularly sensitive to influences from the spatial stimulus arrangement (e.g. saccade amplitude and scanpath length) should be interpreted with caution. Nevertheless, our findings from the comparison of these measures between the TMT-A and TMT-B should not have been driven by differences in the randomized spatial arrangements, because a recent follow-up study provided converging results under conditions where the spatial arrangement of stimuli had been controlled for (see the pre-registration of the project as well as its results at: https://osf.io/fwhjb/)

### Speed vs. accuracy task set influences in the TMT

Next to the addition of eye-tracking measures, this version of the TMT also included a manipulation of participants’ task set, namely their emphasis on either speed or accuracy during task performance. We used this manipulation to compare the variance from the experimental manipulation with the variance due to interindividual differences and examined each test score considering the predominant influence of either experimental manipulation or interindividual variability in each test half. Test scores that primarily reflect the speed-accuracy task set vary more strongly due to the manipulation, whereas test scores that do not primarily reflect this construct vary more strongly due to interindividual variability. In this way, we link test scores with, or we demarcate them from the cognitive construct of the speed-accuracy task set and test whether it is reflected or not reflected in a given test score of the TMT. In the TMT-A, the trial duration, the number of fixations, the number of searching fixations, the eye-hand span, and the scanpath length were dominated by the experimental task manipulation of emphasizing speed or accuracy. As such, the test scores provided by these measures reflect influences of participants’ task set, i.e. their adaptation to current task demands in terms of emphasizing speed or accuracy. Since the trade-off between speed and accuracy of responses affects presumably every task in a large variety of everyday situations [26, 59], it has a high ecological validity. The trade-off also successfully provided a tool to study and explain possible differences for instance in aging [60–62] or the progression of Mild Cognitive Impairment [63]. Therefore, relating the set of test scores to this construct can offer valuable insights into participants’ task performance while practicing the TMT because they provide valid measures of the applied experimental manipulation. On the other hand, we found that fixation durations, saccade amplitudes and the number of guiding fixations were predominantly determined by the interindividual variability in the TMT-A. Furthermore, in the TMT-B all test scores were predominantly determined by the interindividual variability. That is, all of these test scores were not dominated by the current emphasis on speed or accuracy of the participant but rather the variability between participants. In the TMT-A these measures thus represent test scores which are relatively stable across adaptation of participants’ task set of emphasizing speed or accuracy. This confirms previous results on fixation durations and saccade amplitudes [27, 28, 58] and provides divergent validity for their stability in the TMT because of their relative independence from the experimental manipulation. In case of the TMT-B, the relative dominance of interindividual variability speaks to the complexity of the involved functions in the determinants of task. As the trade-off between speed and accuracy is a phenomenon impacting a great number of tasks and processes it presumably depicts a strong effect. Still, variability between individuals in the TMT-B is relatively stronger than this effect. That is not to say, that there are no influences of emphasizing speed or accuracy in the TMT-B, as our previous analyses illustrated, but rather that test scores in the TMT-B are more strongly determined by interindividual variability not caused by the speed accuracy manipulation.

### Enabling a cognitively specific interpretation of the TMT

The new version of the TMT improves the construct validity of the test in two ways. First, the inclusion of additional eye-tracking measures provides test scores directly relatable to specific cognitive functions. Since there is a body of work investigating eye movements with respect to their functions while performing for example manual actions [20] or visual search [64] or influences of cognitive control [21], they can establish connections to such constructs. For instance, the number of fixations and their respective function as searching or guiding, demonstrates that while searching behavior increases in the TMT-B, the guidance of specific actions is constant across test halves. Second, combining the test with an experimental manipulation targeting a specific and established cognitive construct enables the assessment of each test scores with respect to this manipulation. Here, instructing participants to either emphasize their speed or their accuracy during performance allowed us to compare test scores relative to this task set manipulation and evaluate them based on the predominant influence. By comparing the variation in the test scores that is due to the cognitive construct captured by the experimental manipulation (i.e. here the task set for cognitively adjusting speed and accuracy of task performance) with the variation due to interindividual differences we create a measure supporting the test score’s construct validity. That is, test scores that are dominated by the task set manipulation receive a converging construct validation showing that the test score is a valid measure of the construct. At the same time, test scores that are dominated by interindividual variability even despite the experimental manipulation receive a diverging construct validation that demonstrates their relative independence from the construct. This evaluation helps identifying the set of scores best suited for the diagnostic needs. Test scores varying due to the experimental manipulation represent valid measures of the included construct, while test scores varying due to individual differences can be demarcated from the included construct. Taken together using an experimental manipulation in a neuropsychological test as a benchmark for the test scores enables interpretations in light of the experimental construct. Interpretations can be linked to current models and theories of cognitive function and thus improve the understanding of sampled test scores.

### Practical implications

Clinical decisions rely on a clear and differentiated assessment of the neuropsychological functionality of individuals. Based on this assessment conclusions are drawn and treatments are chosen. Extending previous studies on eye-tracking studies in the TMT [31–33], our results reveal how extending neuropsychological tests with eye-tracking supports neuropsychological assessments of cognition. First, eye-tracking measures are easier to interpret in terms of neuro-cognitive functions, because compared with behavioral measures, their neurophysiological underpinnings are better understood [16, 18]. Second, eye movements are often intact even when neurological syndromes impair patients’ manual behavior, so that eye-tracking also enables to cognitively assess patients that would otherwise be untestable due to these impairments [32, 65]. Besides the advantages of eye-tracking for neuropsychological assessments, our findings also show how including experimental manipulations in diagnostic tests supports validation of test scores with respect to the cognitive construct the test aims to assess and increases the conceptual specificity of the test’s scores. That is, one can evaluate the test scores against experimental manipulations that have been established to target a highly specific and theory-grounded cognitive construct. In this way, the high conceptual specificity of the manipulations is transferred to the test scores in question. Here, test scores could be identified as primarily reflecting the executive function underlying the adaptation of performance to speed vs. accuracy demands. For instance, in the TMT-A fixation durations, saccade amplitudes and the number of guiding fixations were dominated by interindividual variability. Thus, their underlying cognitive processes should not be primarily dependent on the task set. This supports and clarifies the interpretation of the scores. Taken together, the here applied approach offers a more mechanistic interpretation of the TMT test scores which helps the diagnostic assessment of executive functions and cognitive impairments, thus supporting clinical decision-making. [32]

## Conclusions

In conclusion, we provide a new version of the TMT comprising additional eye-tracking measures and an experimental manipulation of participants’ task set on emphasizing speed vs. accuracy. This approach allows for a more specific interpretation of test scores in the TMT, going beyond the usually provided compound measures of cognitive functions in the classic test. By including an experimental manipulation into the test, we were able to differentiate between test scores that predominantly reflect the speed-accuracy manipulation vs. the inter-individual variability derived from other cognitive processes. In this way, test scores can be linked to and interpreted considering cognitive theories the manipulation is based on. Using established experimental manipulations in this way, presents a unique approach in including cognitive constructs into tests. Experimental and differential/ correlational approaches in psychology have long been called to move together and benefit from another [66, 67], but only recently this call began to attract answers [68, 69]. The present approach attempts to join this path and suggests a widely applicable way to actively incorporate cognitive theory in neuropsychological settings.

## Supporting information

S1 Table Descriptive results of dependent variables per experimental condition. (DOCX)
