# Correction: Emphasizing speed or accuracy in an eye-tracking version of the Trail-Making-Test: Towards experimental diagnostics for decomposing executive functions

**DOI:** 10.1371/journal.pone.0317043

**Published:** 2025-01-02

**Authors:** The PLOS ONE Staff

There are errors in the Funding statement. The correct Funding statement is as follows: This work is funded by the Deutsche Forschungsgemeinschaft (DFG, German Research Foundation, https://www.dfg.de/)–project number 418552203. It was acquired by R.M.F. and transferred to C.H.P. The funders have no role in study design, data collection and analysis, decision to publish, or preparation of the manuscript.

The Acknowledgment section is not indicated. The Acknowledgment section is as follows: The authors acknowledge support for the publication costs by the Open Access Publication Fund of Bielefeld University and the Deutsche Forschungsgemeinschaft (DFG).

The ORCID iD is missing for the fourth author. Please see the author’s ORCID iD here:

- Author Christian H. Poth’s ORCID iD is 0000-0003-1621-4911
  (https://orcid.org/0000-0003-1621-4911).

The publisher apologizes for the errors.
