# Supplementary material for: Emphasizing speed or accuracy in an eye-tracking version of the Trail-Making-Test: Towards experimental diagnostics for decomposing executive functions
Source: PLoS One. 2022 Sep 12;17(9):e0274579. doi: 10.1371/journal.pone.0274579 (PMC9467318; doi:10.1371/journal.pone.0274579)
Supplement: S1 Table — (DOCX) [file pone.0274579.s001.docx]

**Table S1**

|  | A | | B | |
| --- | --- | --- | --- | --- |
| DV | speed | accuracy | speed | accuracy |
| trial duration  (in sec) | 44.37 (12.78) | 72.55 (23.75) | 81.59 (27.70) | 95.52 (37.97) |
| fixation duration  (in ms) | 163.03 (19.57) | 160.80 (20.75) | 164.53 (21.19) | 165.20 (21.62) |
| saccade amplitude  (in ° va) | 4.41 (0.65) | 4.61 (0.59) | 4.67 (0.64) | 4.43 (0.55) |
| n fixations | 192.27 (67.05) | 267.24 (86.17) | 334.51 (116.91) | 347.54 (129.91) |
| n guiding fixations | 51.15 (11.47) | 53.73 (11.35) | 51.71 (17.82) | 52.46 (13.73) |
| n searching fixations | 52.62 (21.92) | 80.59 (32.19) | 97.14 (41.17) | 113.39 (49.53) |
| eye-hand span  (in ms) | 1,125.13 (314.57) | 1,883.86 (665.94) | 1,582.98 (517.50) | 2,023.66 (895.74) |
| scanpath length  (in pix) | 50,738.74 (17,889.88) | 74,812.30 (23,653.90) | 100,461.86 (38,809.07) | 104,635.70 (40,802.85) |
| Mean values of dependent variables; values in parentheses present standard deviations | | | | |

Descriptive statistics of dependent variables per experimental condition
